# Drug Targets for Cardiovascular-Safe Anti-Inflammatory: In Silico Rational Drug Studies

**DOI:** 10.1371/journal.pone.0156156

**Published:** 2016-06-03

**Authors:** Sajad Shahbazi, Tammanna R. Sahrawat, Monalisa Ray, Swagatika Dash, Dattatreya Kar, Shikha Singh

**Affiliations:** 1 Department of Biotechnology, Punjab University, Chandigarh, India; 2 Centre for System Biology and Bioinformatics, Punjab University, Chandigarh, India; 3 Centre of Biotechnology, Siksha O Anusandhan University, Khandagiri, Bhubaneswar, Odisha, India; University of Buenos Aires, Cardiovascular Pathophysiology Institute, ARGENTINA

## Abstract

Cyclooxygenase-2 (COX-2) plays an important role in memory consolidation and synaptic activity, the most fundamental functions of the brain. It converts arachidonic acid to prostaglandin endoperoxide H2. In contrast, if over-expressed, it causes inflammation in response to cytokine, pro-inflammatory molecule, and growth factor. Anti-inflammatory agents, by allosteric or competitive inhibition of COX-2, alleviate the symptoms of inflammation. *Coxib* family drugs, particularly celecoxib, are the most famous anti-inflammatory agents available in the market showing significant inhibitory effect on COX-2 activity. Due to high cardiovascular risk of this drug group, recent researches are focused on the investigation of new safer drugs for anti-inflammatory diseases. Natural compounds, particularly, phytochemicals are found to be good candidates for drug designing and discovery. In the present study, we performed *in silico* studies to quantitatively scrutinize the molecular interaction of curcumin and its structural analogs with COX-2, COX-1, FXa and integrin αIIbβIII to investigate their therapeutic potential as a cardiovascular-safe anti-inflammatory medicine (CVSAIM). The results of both ADMET and docking study indicated that out of all the 39 compounds studied, caffeic acid had remarkable interaction with proteins involved in inflammatory response. It was also found to inhibit the proteins that are involved in thrombosis, thereby, having the potential to be developed as therapeutic agent.

## Introduction

In recent biomedical research, the utmost task is the effective transformation of simple mechanistic knowledge into clinically effective therapeutics. Now-a-days several drugs have been developed from traditional products and current drug research is keenly investigating the possible therapeutic roles of many medicinal plants and natural products. Prominent among those being examined is turmeric and its main active ingredient is curcumin. Curcumin acts as an antioxidant, anti-inflammatory, anticarcinoma, antimicrobial, antiviral, hypoglycemic and wound healer. It has shown the therapeutic ability in numerous diseases and in several kinds of cancer *in vitro* and *in vivo*. Inflammation is essential for the removal of challenges to the organism and successive repair of homeostasis [1]. However, incorrect inflammation is associated with pathological conditions such as sepsis, trauma, inflammatory bowel diseases, chronic wounds, rheumatologic disorders and asthma; various other diseases, such as cancer, diabetes, atherosclerosis, Alzheimer’s, and obesity are also associated with dysregulated inflammation. Inflammation is therefore a reasonable drug target. Nevertheless, one feature that is often ignored in the drug development process is that drug candidates, whether aimed at modulating inflammation or other processes, may have unforeseen effects *in vivo* based on their effects on the inflammatory response (e.g. gastrointestinal toxicity of cyclooxygenase-2 inhibitors [2, 3]. Cyclooxygenase (COX) or prostaglandin (PG) H synthase, is the key enzyme in inflammation scenario. Two isoforms of this enzyme COX-1 and COX-2, directly participate in prostanoids synthesis pathway. COX-1 and COX-2 are expressed under normal conditions in the human body and have physiological and immunological activity in some tissues such as cytoprotection of gastrointestinal tissues and platelet aggregation by COX-1, fundamental function of the brain by COX-2 and immunoreactivities in brain by both COX-1 and COX-2 [4]. However, beside vital activity in the brain, COX-2 plays an important role in the inflammation scenario in response to cytokines and pro-inflammatory molecules.

Following the characterization of COX-2, in 1990s, most of the inflammation research was focused on the role of COX-2 in inflammation and its inhibition [5, 6, 7]. A new generation of selective non-steroidal anti-inflammatory drugs (NSIADs) superseded non-selective anti-inflammatory drugs such as aspirin and traditional NSIADs. Through the inhibition of COX, both groups of drugs can alleviate the symptoms of inflammation but selective NSAIDs, beside their brilliant anti-inflammatory function, cause increase the rate of thromboxane A2 (TXA2) in the body which results in vasoconstriction, vascular proliferation, platelet aggregation and thrombosis. Due to cardiovascular risks of the selective NSAIDs and other side effects, most recent research focuses on natural products because of fewer side effects and in some cases, significantly beneficial results like GSPE and garcinia extract in treatment of ulcerative colitis [8]. Among phytochemical compounds extracted from different medicinal plants, Curcumin, from Curcuma longa, by binding to COX-2, intervenes in prostaglandin pathway and inhibits catalysis of arachidonic acid to PGH2. Also curcumin, by binding to the COX-1 active site, inhibits the production of thromboxane A2 consequently, can reduce the risk of thrombosis [9]. Curcumin can inhibit COX-2 in allosteric or competitive process. COX-1, Coagulation factor Xa (FXa) and integrin (α_IIb_β_3)_ are most well-known enzymes, which are directly or indirectly participating in thrombosis pathways [8, 9]. The active form of FXa, plays an important role in the thrombosis by both extrinsic and intrinsic pathways [8].

Integrin α_IIb_β_3_ receptor, glycoprotein IIb/IIIa, is an integrin-complex receptor for fibrinogen on platelets and plays a crucial role in thrombosis by platelet aggregation and adhesion to the sub-endothelium [10, 11]. GP IIb/IIIa receptors are present in large numbers on the surface of each platelet and due to being specific for platelets; their inhibition only impedes platelet aggregation without any effect on platelet adhesion. Therefore, it eliminates the risk of thrombosis and ischemic damage in hemostasis [12, 13].

Due to the high cardiovascular risk of NSAIDs, it is necessary to find an adjuvent or replacement compound/s to reduce the cardiovascular risk of this family of drug. To reach this goal, we selected phytochemicals as a major source for drug designing. But due to lack of comprehensive data regarding efficacy, ADME, toxicity and multi-targeting information, requires extensive *in silico* study on the effect of curcumin and its analogs on inflammation and their efficacies on cardiovascular system combined with ADMET study of these bioactives. The present study has been undertaken to study the interaction of curcumin and its analogs with the enzymes involved in inflammation and thrombosis and investigate AMDET of these compounds to obtain potential therapeutic candidate/s for further studies to develop cardiovascular-safe anti-inflammatory-medicine (CSAIM).

## Materials and Methods

### Selection of proteins

The structures of COX isoforms—COX-1 (PDB Id- 3N8Y) and COX-2 (PDB Id- 1CVU & 3LN1), Coagulation factor Xa (FXa) (PDB Id-1IQM) and integrin αIIbβ3 (PDB Id-3FCU) were obtained from the RCSB Protein Data Bank (http://www.rcsb.org/pdb/) with X-ray diffraction resolutions in 3.00, 2.40, 2.40, 2.60 and 2.90 angstroms respectively.

### Preparation of proteins and ligands

Preparation of the retrieved protein was performed by using protein Preparation Wizard of Schrodinger suite 2011 (Schrödinger Suite; Epik version 2.2; Impact version 5.7; Prime version 2.3, Schrödinger, LLC, New York, NY, 2011). The energy minimization / geometrical optimization of target proteins have been done via OPLS 2005 forcefield with RMSD as 0.30.

**Fig 1:**
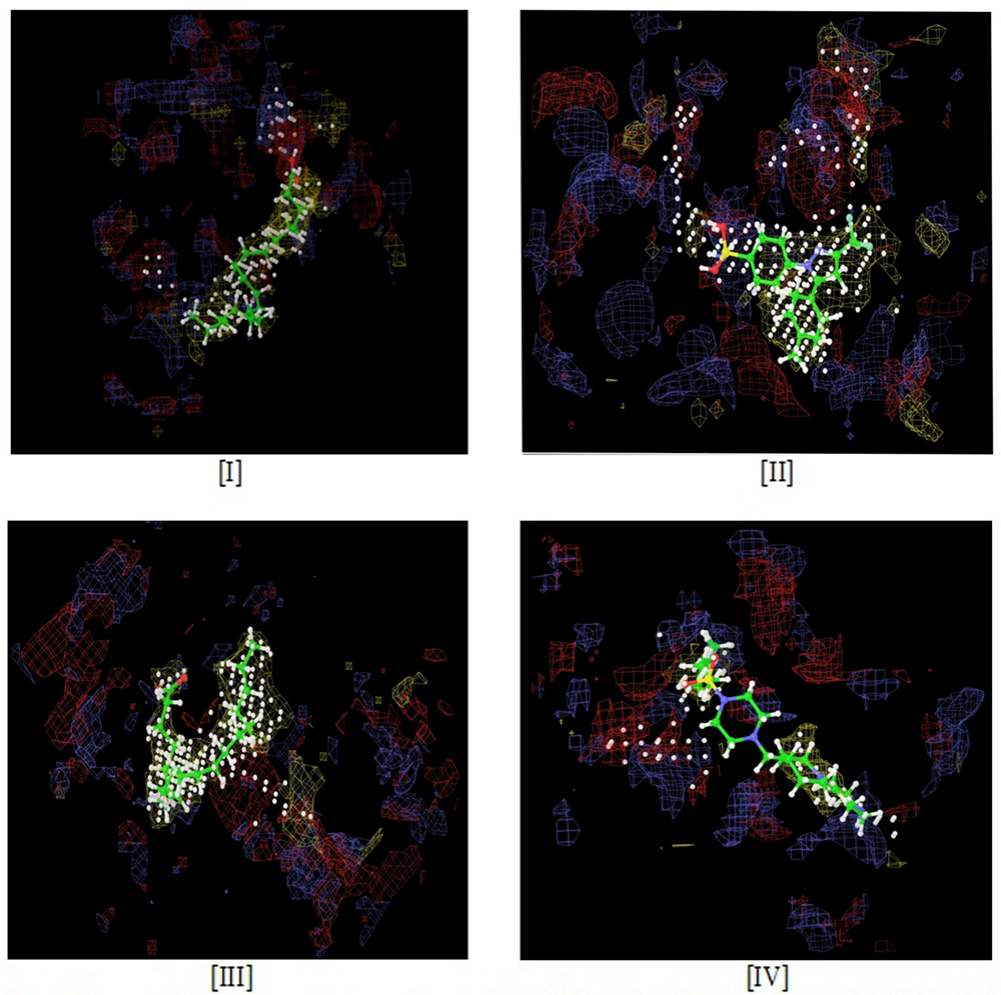
Sitemap of 4 receptors and their own ligands. [I]. COX-1, [II]. COX-2, 3LN1, [III]. COX-2, 1CVU, [IV]. FXa.

Binding site characterization of the processed protein was performed by using SITEMAP 2.5 module (Schrödinger, LLC, New York, NY, 2011) based on the chemical possession of amino acids such as hydrophobicity, hydrophilicity, and metal binding regions of 3 receptors COX-1, COX-2 (1CVU, 3LN1) and FXa which are depicted in Fig 1 with respect to the included ligands.

**Fig 2:**
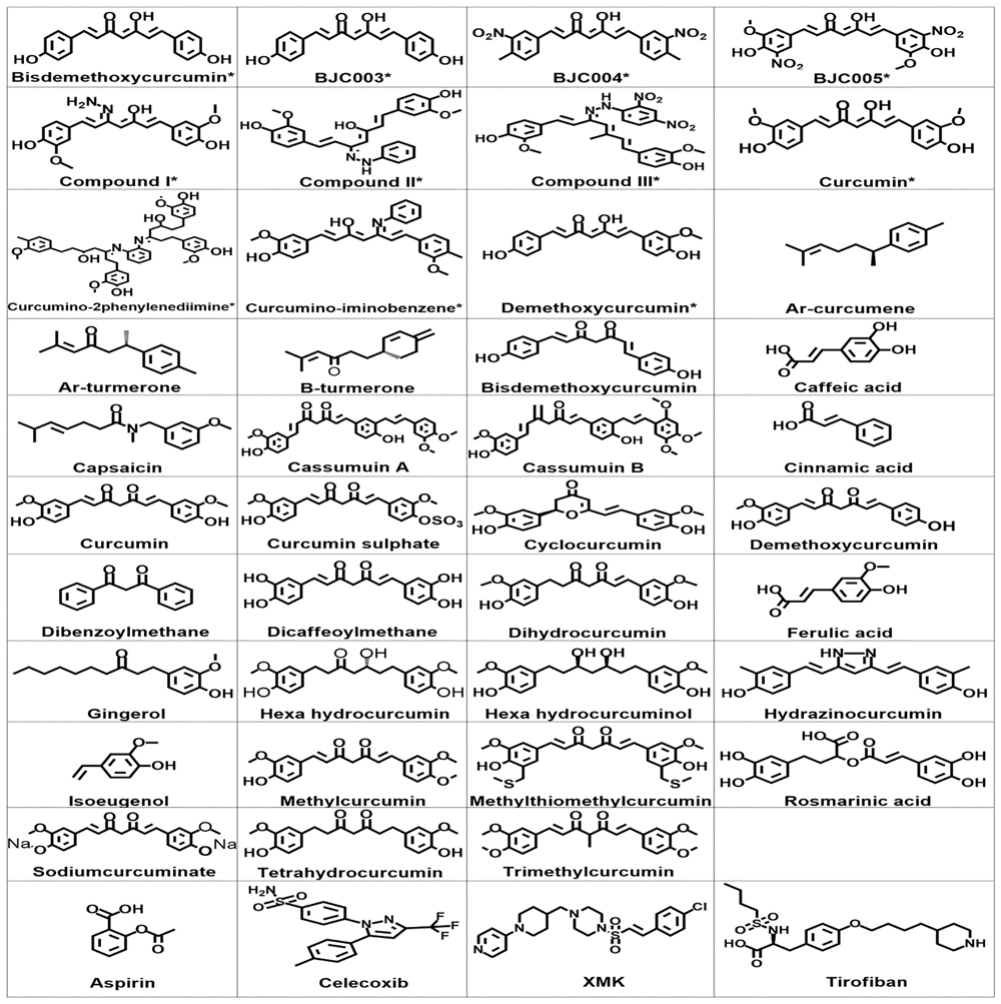
2D chemical structures of compounds.

Preparation of ligands was done by using “LigPrep 2.5” module of Schrodinger Suite 2011 using the OPLS forcefield 2005 at biologically relevant pH by assigning the protonation states include disconnecting of group I metals in simple salts, deprotonating strong acids and protonating strong bases, while adding explicit hydrogens and topological duplicates. The 2D structure and molecular properties of all compounds are given in Fig 2 and Table 1, respectively.

**Table 1:** Molecular properties of all curcumin analogs via QikProp 3.4 2011.

Title	mol _MW	Donor HB	Accpt HB	QPlog PC16	QPlog Poct	QPlog Pw	QPlog Po/w
**Enol**							
bisdemethoxycurcumin	308.33	2	5.50	12.24	17.32	11.06	2.55
BJC003	304.39	0	4.00	11.65	14.59	6.29	4.52
BJC004	394.38	0	6.00	13.60	17.87	8.57	3.13
BJC005	458.38	2	9.00	14.88	23.29	13.73	1.76
Compound I	382.42	5	5.25	14.51	22.46	13.96	2.91
Compound II	458.51	4	5.75	17.31	24.70	13.81	5.24
Compound III	546.54	3	7.00	18.69	26.05	13.62	4.63
Curcumino-2- phenylenediimine	820.98	6	11.40	28.93	41.73	21.14	7.97
Curcumino-iminobenzene	443.50	3	4.75	16.57	22.57	11.32	5.60
Curcomin	368.39	2	7.00	13.08	19.26	11.66	2.69
Demethoxycurcumin	338.36	2	6.25	12.58	19.14	11.35	2.58
**Keto**							
Ar-Curcumene	202.34	0	0.00	7.21	8.06	0.11	6.33
Ar-Turmerone	216.32	0	2.00	7.32	9.22	3.13	3.63
B-Turmerone	218.34	0	2.00	7.43	9.35	2.82	3.88
Bis-demethoxycurcumin	308.33	2	5.50	12.21	16.82	11.10	2.47
Caffeic acid	180.16	3	3.50	7.04	12.64	9.87	0.56
Capsaicin	275.39	0	3.75	9.17	12.17	5.46	3.83
Cassumuin A	514.57	2	7.75	18.30	24.79	12.64	5.45
Cassumuin B	544.60	2	8.50	19.04	25.74	12.96	5.58
Cinnamic acid	148.16	1	2.00	6.04	8.37	5.69	1.91
Curcumin	368.39	2	7.00	12.80	18.75	11.45	2.92
Curcumin sulphate	448.44	2	10.75	15.11	23.60	15.54	2.01
Cyclocurcumin	368.39	2	5.75	12.59	19.15	11.28	3.33
Demethoxycurcumin	338.36	2	6.25	12.47	18.68	11.26	2.65
Dibenzoylmethane	224.26	0	1.75	8.76	9.78	4.66	3.55
Dicaffeoylmethane	340.33	4	7.00	13.39	21.69	15.30	1.19
Dihydrocurcumin	370.40	2	7.00	12.72	19.07	11.18	3.09
Ferulic acid	194.19	2	3.50	6.90	11.43	8.03	1.38
Gingerol	278.39	1	3.50	9.38	12.41	4.75	4.18
Hexa hydrocurcumin	374.43	2	5.70	12.99	18.01	9.62	3.72
Hexa hydrocurcuminol	376.45	4	6.40	12.99	20.73	12.73	3.06
Hydrazinocurcumins	332.40	3	2.50	12.90	18.36	9.62	4.33
Isoeugenol	150.18	1	1.50	5.38	7.03	4.29	1.91
Methylcurcumin	396.44	0	7.00	12.88	17.01	8.04	4.11
Methylthiomethylcurcumin	488.61	2	8.00	14.79	21.87	10.84	4.24
Rosmarinic acid	374.35	5	7.00	13.99	24.26	16.77	1.38
Sodiumcurcuminate	368.39	2	7.00	12.92	19.03	11.55	2.80
Tetra hydrocurcumin	372.42	2	4.75	12.63	17.45	8.83	4.18
Trimethylcurcumin	410.47	0	7.00	12.88	17.51	7.89	4.39

**mol_MW** = Molecular weight: recommended value(R.V.):130–725, **donorHB** = Estimated number of hydrogen bonds that would be donated by the solute to water molecules in an aqueous solution: R.V.:0.0–6.0);**accptHB** = Estimated number of hydrogen bonds that would be accepted by the solute from water molecules in an aqueous solution: R.V. = 2.0–20.0;**QPlogPC16** Predicted hexadecane/gas partition coefficient: R.V. = 4.0–18.0; **QPlogPoct**‡ = Predicted octanol/gas partition coefficient: R.V. = 8.0–35.0; **QPlogPw** = Predicted water/gas partition coefficient: R.V. = 4.0–45.0; **QPlogPo/w** = Predicted octanol/water partition coefficient: R.V. = –2.0–6.5.

### ADMET studies

ADMET study has been performed by employing of two strong analytical softwares: Schrodinger Suite 2011 and online TOPKAT approaches of Accelrys Environmental Chemistry and Toxicology Workbench, Accelrys Inc., San Diego, USA, (https://ect01.accelrysonline.com/webport/ECT/main.htm). Through “Qikprop 3.4” module of Schrodinger Suite 2011 [14], the pharmacokinetic profiles of the compounds were assessed by #start parameter, as an overall ADMET-acceptance score for the drug likeness parameter, which indicates property descriptors out of range of values optimized for 95% of known drugs through Jorgensen criteria [15]. These criteria includes: SASA/Smol (300−1000), FOSA(0−750), FISA (7−330), total solvent-accessible volume (volume), PISA (0−450), Glob (0.75−0.95 for 95% of drugs), number of likely metabolic reactions (Metab; 1−8 for 95% of drugs), QPlogKhsa (-1.5−1.5), molecular weight (mol_MW), donorHB, accptHB, QPlogHERG (concern,<-5), QPPMDCK(nm per sec; <25 poor, >500 great), QPlogKp (-8−-10), partition coefficient including QPlogPo/w (octanol/water), QPlogPw (water/gas), QPlogPoct‡ (octanol/gas) and QPlogPC16 (hexadecane/gas) [15, 16, 17], central nervous system activity (CNS) [18], QPlogBB (-3−1.2) [19], QPPCaco (<25 poor, >500 great) [20], the human oral absorption level, the maximum transdermal transport rate (Jm; K_p_ X MW X S; μg cm^-2^hr^-1^),PM3 calculated ionization potential (IP(eV); 7.9−10.5), PM3 calculated electron affinity (EA(eV); -0.9−1.7), and the number of violations of Lipinski’s rule of five [16,21] of the various curcumin analogs. TOPKAT features provide a means to accurately assess the toxicity of compounds such as mutagenicity (Ames test v3.1), rodent carcinogenicity from the FDA dataset for both female and male (v3.1), skin sensitization (GPMT) (v.6.1), skin irritancy (v6.1), ocular irritation (v5.1), weight of evidence (WOE) (v5.1), aerobic biodegradability (v6.1), EC50, LD50 [22]. The ADME and toxicity results of all compounds are listed in Table 1, S1 and S2 Tables, respectively. The ADME profiles of the 15 selected compounds are presented in Table 2.

**Table 2:** ADME profiling.

Title	QPlog S	CIQPlog S	QPlog HERG	QPP Caco	QPlog BB	QPP MDCK	QPlog Kp	QPlog Khsa	HOA	RF	RT	CNS	#metab	Jm
**Enol**														
Bisdemethoxycurcumin	-4.04	-4.00	-6.48	160.10	-2.02	68.29	-2.80	-0.05	3	0	0	-2	3	0.05
BJC003	-5.50	-4.40	-6.52	1468.98	-0.86	749.68	-1.23	0.50	3	0	0	-1	3	0.06
BJC004	-5.33	-5.39	-6.38	37.28	-2.90	14.14	-4.42	0.26	3	0	0	-2	5	0.00
**Keto**														
Ar-Turmerone	-4.02	-3.10	-4.26	4390.44	-0.01	2448.08	-1.38	0.40	3	0	0	0	5	0.86
B-Turmerone	-3.99	-2.95	-4.14	4078.42	-0.10	2260.58	-1.41	0.41	3	0	0	0	5	0.86
Bisdemethoxycurcumin	-3.90	-4.00	-6.40	144.14	-2.04	60.97	-2.87	-0.07	3	0	0	-2	3	0.05
Caffeic acid	-1.34	-1.84	-2.21	22.13	-1.56	10.23	-4.52	-0.80	2	0	1	-2	2	0.25
Capsaicin	-4.41	-2.90	-3.83	5079.75	-0.05	3794.09	-0.71	0.09	3	0	0	0	5	2.10
Cinnamic acid	-1.66	-1.78	-2.41	203.75	-0.56	112.72	-2.56	-0.51	3	0	0	-1	0	6.34
Cyclocurcumin	-5.17	-5.31	-6.00	330.89	-1.47	149.69	-2.76	0.35	3	0	0	-2	6	0.00
Dibenzoylmethane	-3.57	-3.74	-5.62	1974.58	-0.40	1032.11	-1.02	0.23	3	0	0	0	0	5.79
Ferulic acid	-1.91	-2.15	-2.27	62.62	-1.19	31.49	-3.70	-0.61	3	0	0	-2	2	0.48
Hydrazinocurcumins	-5.84	-5.69	-6.54	411.89	-1.49	189.66	-2.30	0.67	3	0	1	-2	4	0.00
Isoeugenol	-1.57	-1.97	-3.58	3370.25	-0.01	1839.46	-1.58	-0.25	3	0	0	0	2	58.62

**CNS**: central nervous system activity-2, -1, 0, 1, 2: -2 = completely inactive, -1 = very low activity, 0 = low activity, 1 = medium activity, 2 = completely active, 3 = high, **Jm**: maximum transdermal transport rate, **Metab**: Number of likely metabolic reactions; 1–8, **QPlogS**: prediction aqueous solubility level; recommended range -6.5<x<0.5, **CIQPlogS**:Conformation-independent predicted aqueous solubility; -6.5<x<0.5,**QPlogHERG**: Predicted IC50 value for blockage of HERG K+ channels; <-5 = concern, **QPPCaco**: Predicted apparent gut-blood barrier permeability; <25 = poor, >500 = great, **QPlogBB**: Predicted brain/blood partition coefficient; –3.0–1.2, **QPPMDCK**: Predicted apparent MDCK cell permeability; <25 = poor,>500 = great, **QPlogKp**: Predicted skin permeability; range = -8<x<-1,** QPlogKhsa**: Prediction of binding to human serum albumin; –1.5–1.5,**HOA**: human oral absorption level;1, 2, 3; 1 = low, 2 = medium,**RF**: the number of violations of Lipinski’s rule of five, **RT:** the number of violations of Jorgensen’s rule of three.

### Receptor—ligand interactions

Docking studies were performed by using “Glide 5.7” (Grid-Based Ligand Docking with Energetics) module in Extra Precision (XP) mode [23, 24, 25] and the molecular mechanics/Generalized Born Surface Area (MMGBSA) [26,27] for interaction of each complex of ligand-protein has been calculated via Prime 3.0 application of Schrodinger Suite 2011. The results of docking and MMGBS are given in Table 3.

**Table 3:** Docking scores and MMGBS properties.

Title	COX-1		COX-2				FXa		Integrin α2bβ3	
	3N8Y		1CVU		3LN1		1IQM		3FCU	
	XP GScore*	DG*	XP GScore*	DG*	XP GScore*	DG*	XP GScore*	DG*	XP GScore*	DG*
**Enol**										
Bisdemethoxycurcumin	-6.14	-32.66	-9.72	-83.65	-8.87	-58.87	-6.28	-67.37	-2.59	-27.14
BJC003	-5.15	-49.90	-11.45	-91.26	-7.56	-56.29	-5.61	-70.78	0.54	-41.43
BJC004	-5.17	-62.81	-6.84	-61.16	-6.69	-82.20	-5.73	-59.35	-0.03	-47.09
BJC005	-5.17	-37.97	-4.43	-46.20	-2.77	-35.97	-5.23	-50.50	-3.42	-33.89
Compound I	—	—	-6.50	-43.51	-3.66	-32.83	-5.97	-71.05	-1.63	-46.23
Compound II	—	—	-5.53	-54.62	-3.47	-39.92	-4.96	-55.38	-2.07	-25.11
Compound III	—	—	-7.13	-57.83	2.11	-17.56	-3.57	-64.96	3.77	-22.95
Curcomin	-7.31	-49.76	-10.51	-76.95	-7.86	-66.94	-7.30	-60.82	-3.20	-31.39
Curcumino-2-phenylenediimine	—	—	-9.79	-82.29	-8.96	-46.31	—	—	-3.23	-42.28
Curcumino-iminobenzene	—	—	-7.39	-53.44	-4.80	-37.49	-6.87	-67.24	-2.16	-43.48
Demethoxycurcumin	-6.64	-42.33	-9.94	-75.62	-5.97	-34.01	-6.88	-65.99	-3.11	-34.70
**Keto**										
Ar-Curcumene	-7.24	-53.66	-6.82	-60.42	-5.94	-67.89	-4.28	-58.60	0.93	-23.27
Ar-Turmerone	-7.68	-59.41	-7.41	-63.84	-8.14	-71.76	-4.87	-44.75	-1.28	-24.07
B-Turmerone	-7.19	-60.80	-6.96	-59.70	-6.71	-78.03	-4.80	-45.21	-0.64	-27.54
Bis-demethoxycurcumin	-5.74	-37.71	-9.59	-83.06	-8.15	-52.01	-6.75	-55.27	-2.51	-27.43
Caffeic acid	-6.35	-19.37	-6.52	6.07	-5.77	-18.67	-4.78	-30.26	-3.60	-10.11
Capsaicin	-5.66	-26.85	-7.08	-78.49	-6.98	-59.92	-5.18	-55.10	-0.09	-20.79
Cassumuin A	-4.30	-49.64	-6.32	-35.69	-9.00	-79.50	-5.16	-72.52	-2.21	-53.39
Cassumuin B	—	—	-7.80	-50.41	-7.46	-57.10	-5.91	-84.73	-2.84	-21.36
Cinnamic acid	-5.41	-17.10	-5.45	-22.49	-5.53	-24.73	-3.44	-25.80	-1.61	-7.73
Curcumin	-6.64	-49.43	-7.53	-51.60	-9.23	-64.40	-7.20	-67.37	-3.33	-31.44
Curcumin sulphate	-4.88	-51.25	-4.28	-48.53	-4.04	-43.04	-5.08	-44.49	-2.85	-28.00
Cyclocurcumin	—	—	-6.38	-39.94	-4.61	-37.57	-6.98	-76.40	-2.87	-17.46
Demethoxycurcumin	-6.53	-46.23	-9.73	-85.70	-7.28	-40.49	-6.88	-49.34	-3.06	-31.77
Dibenzoylmethane	-8.25	-56.25	-7.82	-65.87	-8.90	-60.83	-4.29	-33.86	0.35	-26.34
Dicaffeoylmethane	-6.60	-31.81	-6.07	-47.49	-8.73	-50.14	-7.55	-42.76	-4.11	-31.47
Dihydrocurcumin	-7.26	-48.64	-7.07	-39.88	-4.80	-37.94	-8.60	-68.08	-3.27	-15.05
Ferulic acid	-5.76	0.71	-6.62	-10.20	-5.86	-25.38	-4.32	-30.91	-2.54	-20.02
Gingerol	-6.23	-43.69	-7.00	-62.27	-7.34	-81.37	-6.52	-65.49	-1.70	-36.98
Hexa hydrocurcumin	-8.09	-50.29	-7.85	-52.53	-4.89	-36.97	-8.49	-82.25	-3.49	-31.30
Hexa hydrocurcuminol	-9.11	-57.58	-11.99	-90.05	-8.28	-24.14	-9.78	-85.81	-3.59	-41.71
Hydrazinocurcumins	-5.91	-45.54	-3.48	-40.42	-2.35	-29.22	-4.95	-51.76	-0.76	-35.26
Isoeugenol	-5.89	-38.78	-5.74	-40.18	-5.31	-45.47	-4.99	-44.85	-2.62	-28.11
Methylcurcumin	-5.74	-57.93	-5.89	-56.15	-6.81	-76.28	-3.98	-46.72	-1.76	-37.90
Methylthiomethylcurcumin	-5.88	-76.43	-6.85	-57.11	-2.27	-46.24	-6.35	-65.92	-1.83	-36.71
Rosmarinic acid	-6.48	-34.57	-8.90	-29.80	-7.63	-44.33	-9.85	-65.53	-3.08	-24.97
Sodiumcurcuminate	-5.96	-43.32	-7.15	-51.43	-6.92	-63.94	-6.89	-71.17	-1.82	-48.83
Tetra hydrocurcumin	-9.12	-72.00	-8.29	-48.59	-7.14	-50.20	-8.77	-72.59	-2.29	-32.51
Trimethylcurcumin	-3.60	-46.53	-5.41	-41.36	-7.08	-66.89	-3.61	-55.00	1.21	-50.82
**Control**										
Aspirin	-5.21	-22.40	—	—	-4.43	-23.66	—	—	—	—
Celecoxib	—	—	—	—	-11.71	-89.33	—	—	—	—
TIROFIBAN	—	—	—	—	—	—	—	—	-5.23	-10.88
XMK	—	—	—	—	—	—	-6.29	-96.04	—	—

*DG (ΔGbind) = Gcomplex– (Gprotein + Gligand) where ΔGbind is Ligand binding energy;

**XP Gscore**: Extra Precision Glide score.

### Visualization of interaction between selected candidate/s and residues in receptors

After selection of potent molecule/s through ADMET filtering and the affinity values investigation, the complex of receptor-ligand was mapped via XP visualizer approaches of Schrodinger 2011 and the receptors surfaces were configured based on the electrostatic potential of residues in binding packet of protein by truncating the receptor surface in 5 Å from ligand with 20% transparency.

### Selection of cardiovascular-safe anti-inflammatory compound/s

Compounds that have been selected possess ADMET profiles within the recommended range for each criterion and highest affinity values for inflammation responsible receptors with PDB IDs:1CVU & 3LN1. Then qualified compounds have been considered for the cardiovascular safety via study of the potential inhibitory level of such compounds for the thrombosis responsible enzymes, COX-1, FXa and integrin αIIbβ3/GP αIIbβ3 with PDB IDs: 3N8Y, 1IQM and 3FCU respectively. Finally, the potential therapeutic compounds will be selected.

## Result and Discussion

### Curcumin analogs

Curcumin, a linear di-arylheptanoid, contains two oxy-substituted aryl moieties linked through unsaturated linear seven-carbon chain (Fig 2). Curcumin analogs are classified into groups, enol and keto, based on their chemical structures. Enol derivatives are synthetic while keto members, almost all are natural. The most natural substituents are of the oxy type, such as hydroxy or methoxy elements, like bisdemethoxycurcumin (BDMC) and demethoxycurcumin (DMC), which differ in methoxy substitution on the aromatic ring [28]. Most of naturally compounds used in this study have some structural similarity to the curcumin molecule containing at least one aryl function with 3, 4 substitutions, either a methoxylated phenol/ catechol, or various metabolites of curcumin. These include ferulic acid, cinnamic acid, caffeic acid, chlorogenic acid, capsaicin, gingerol, dibenzoylmethane, dehydrozingerone, cassumuin, dihydrocurcumin (DHC), tetrahydrocurcumin (THC), hexahydrocurcumin (HHC), octahydrocurcumin (OHC), curcumin glucuronide, and curcumin sulfate.

### Protein and ligand preparation

The structures of COX1, COX2, FXa and integrin αIIbβIII were downloaded from PDB (http://www.rcsb.org/pdb/) and analyzed with Schrödinger 2011 to predict the binding sites for the ligands. The ligands were prepared using Schrödinger 2011 and their molecular properties have been computed via Qikprop 3.4 (Table 1).

### ADME prediction

**Fig 3:**
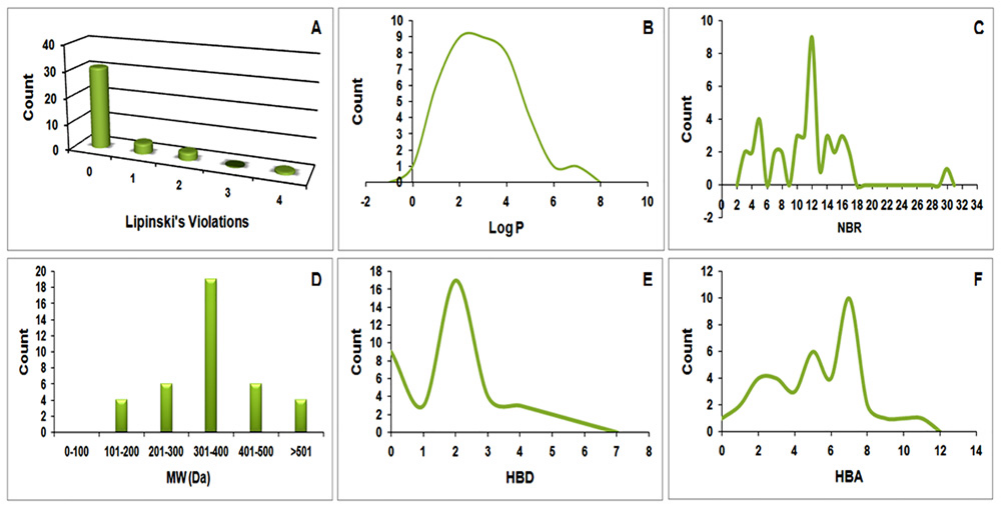
Drug-likeness criteria distribution graphs. (A, D) The frequencies histogram of Lipinski violations and molecular weight and (B, C, E, F) the log P, NRB, HBA, and HBD, distribution curves respectively for Curcumin and analogs respectively.

It is crucial to check the quality of ligands in terms of oral and intenstinal absorption level like the ability of ligands to distribute through the blood stream, level of metabolism, and the ability of excreation from the body besides their toxicity profiling, to consider the ligands as drugs. The oral availabilities of the compounds have been evaluated by the use of Lipinski’s criteria [19, 28]. Fig 3, shows the distribution of MW, HBD, HBA, log P and NRB of bioactives which are used to assess the oral availability. NRB was added as one of the ro5 criteria for natural products due to the wide range of conformationally flexibility of bioactives to consider the desired pharmacokinetics and drug metabolism as suggested by Ntie-Kang, et al. (2013) [29]. According to the results of Lipinski’s ro5, 79.5% of the compounds had no violation and 89.76% of the compounds having less than 2 violations (Fig 3A). The logP distribution with a Gaussian shape curve showed maximum frequency of 9 at logP values of 2 and 3 units (Fig 3B). NBR curve also showed fluctuation in distribution of compounds with a peak at 12 and maximum frequency of 9 (Fig 3C) which indicates the degree of flexibility of the compounds. The molecular weight distribution graph showed the peak between 301–400 Daltons (Fig 3D). The HBA and HBD distribution curves indicate that 10 compounds showed the highest hydrogen bond acceptor value of 7 while 17 compounds showed the acceptable hydrogen bond value of 2 (Fig 3E and 3F). The MW distribution indicated that most of the compounds are within drug-like range except 7.69% compounds having molecular weight >500.

**Fig 4:**
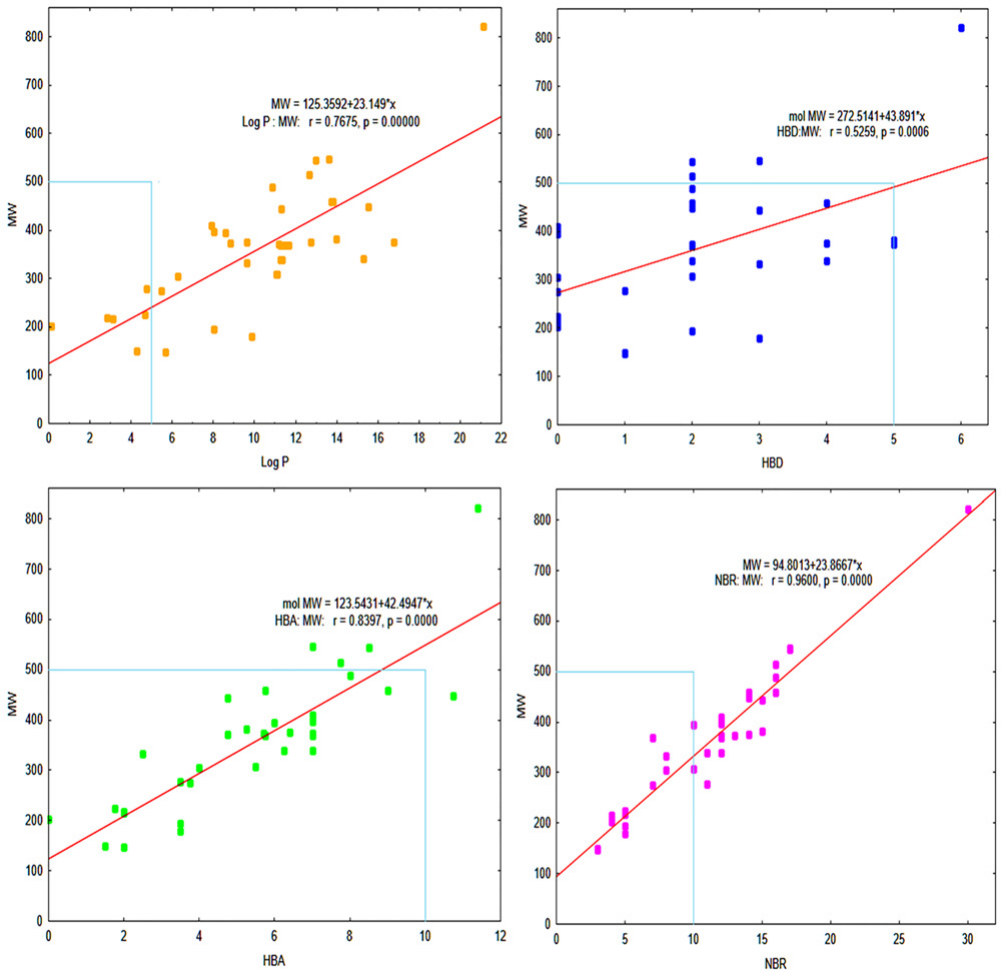
The scatter plot of correlation between molecular weight (MW) and 4 molecular descriptors: (A) The distribution of the calculated log P versus MW, (B) HBD against MW, (C) HBA against MW, and (D) NRB versus MW.

Fig 4 shows scatter plots of the mutual relationship between the MW and the other calculated parameters (HBD, HBA, log P and NRB) and indicated the highest densities of points within the Lipinski compliance regions (MW<400, 0.5<log P<5, HBA≤5.5, HBD≤3) for which NRB≤10. The Lipinski’s criteria data for all compounds listed in Table 1 and S2.2 Table. Therefore, 38.4% out of total compounds studied, i.e. 14 compounds possessed drug—likeliness properties which are listed in Table 2.

**Fig 5:**
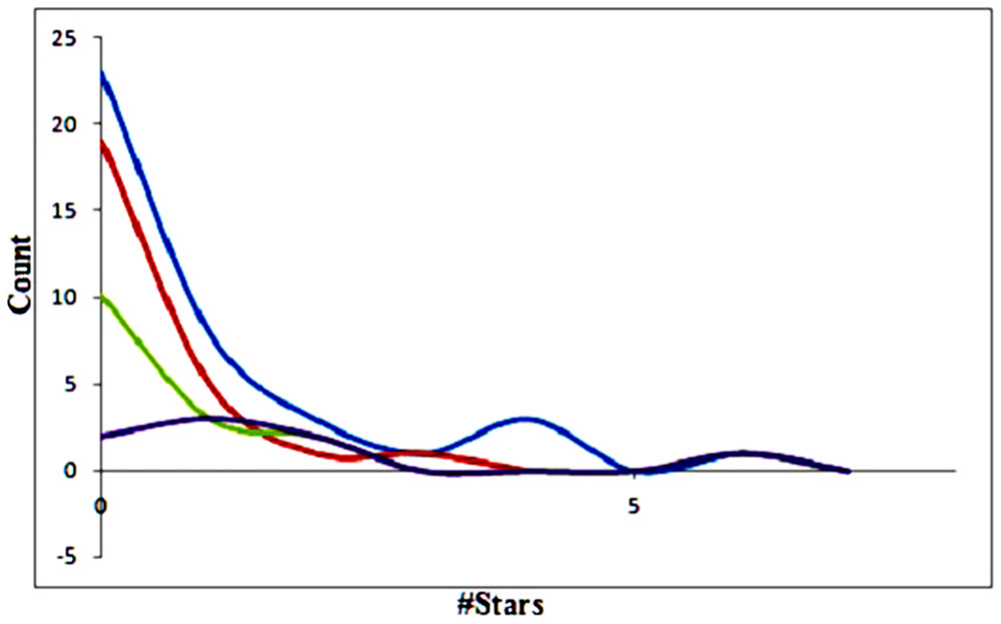
The #stars distribution curves of curcumin and analogs (blue line), with 3 standard subsets: drug-likeness = red, lead-likeness = green, and fragment-likeness = violet.

To investigate drug metabolism and pharmacokinetics, QikProp software was used that employs around 24 molecular descriptors to determine the #star parameter. The recommended values for #star by Schrodinger are in between the range 0–5 in which 1 indicates that the computed property of a molecule is out of the range for 95% of known drugs. Fig 5 shows the distribution of #stars among the compounds in which the #stars parameter on the X-axis and count numbers are on the Y-axis. Count numbers are based on the standard drug-like (MW<500; log P<5; HBD≤5; HBA ≤10), lead-like (150≤ MW ≤350; log P≤4; HBD ≤3; HBA ≤6) and fragment-like (MW ≤250; 22≤ log P≤3; HBD<3; HBA<6; NRB<3) criteria. Out of all the compounds studied the acceptable range for drug—likeliness, lead-likeliness and fragment likeliness of #star = 0 was 73.08%, 62,5% and 25% while #star ≤2 was 96.12%, 93.75% and 87.5% respectively. The compounds having #star = 0 were further studied as they are more likely to have therapeutic potential.

### Bioavailability

The bioavailability of each compound can be determined through two processes, absorption and the first-pass metabolism of the liver. Absorption can be considered via several factors such as solubility, the ability of the compound to pass through the gut wall which depends on the permeability of the compound, the ability of compound to interact with shuttles in the gut wall like, transporters and metabolizing enzymes. The metabolism depends on the functional groups in the compound structure. The oral absorption computed by the Jorgensen’s famous “Rule of Three” (ro3) parameters which are known as the likelihood of the oral availability and include log S>-5.7, QPPCaco>22 nm/s and # primary metabolites<7. Other important parameters for prediction of bioavailability include the predicted of human oral absorption percentage, the predicted qualitative human oral absorption, and the conformation-independent predicted aqueous solubility, CIlog S which are calculated based on the similarity of compounds with their close analogs which are experimentally tested and QikProp uses these data set to predict the solubility models. For similarity>0.9, the predicted property by QikProp is countable via adjusted formula given in the equation P_pred_ = SP_exp_+(1-S)P_QP_, where S is the similarity, P_exp_ and P_QP_ are the respective experimental and QikProp predictions for the most similar molecule within the training set. Log S and #metab are the parameters for prediction of aqueous solubility levels and number of likely metabolic reactions respectively. The Caco-2 model is a parameter for predicting the gut blood barrier permeability which is a non-active transport in nm/s. However, it is often an inaccurate computationally prediction, but can help to understand and estimate approximate transportability values of compounds through the gut wall [29]. The permeability prediction depends on the molecular properties such as the size, flexibility which is dependent on the NRB [29], overall lipophilicity, shape, and the capacity to make hydrogen bonds. The results given in Table 2 indicate 14 compounds were in the recommended ranges of QPPCaco and #metab. Except Hydrazinocurcumins [30] all other 13 compounds had an acceptable value of log S and except caffeic acid [31], all showed high level of oral absorption based on the common parameters of the ro3 such as CIlog S, percentage of oral absorption and qualitative predicted oral absorption.

### The prediction of blood/brain penetration (QPlogBB)

The BBB partition coefficient was estimated to predict the blood brain barrier permeability for each compound and accessibility of bioactives for central nervous system. The polarity of compounds is inversely proportional to the BBB penetration. However to predict the BBB penetration other parameters such as the CNS activity, logB/B and MDCK have to be considered. The predicted CNS activity with a score range from -2 (inactive) to +2 (active) showed that none of the compounds were active in the CNS predicted value >1. The predicted values of logB/B indicated that all 13 compounds selected previously were in the acceptable range (-3.0 to 1.2). QPPMDCK is an additional criterion which is used to predict BBB penetration. This parameter is used for non-active transportation through the Madin-Darby canine kidney (MDCK) due to expression of transporter protein and fewest number of metabolism enzymes. Therefore, it is considered as a desirable parameter to mimic the BBB penetration and also mostly used for oral absorption. The result showed that nearly 46.15% out of 13 compounds had values within the acceptable range of 25 to 500 nm/s.

### The prediction of dermal penetration

The logK_p_ known as the skin permeability parameter is used to predict the penetration of drugs/compounds through the skin. The logK_p_ values showed that, except the capsaicin rest of the 13 compounds laid within the recommended range of 95% of known drugs of dermal penetration. For the maximum trans-dermal transport rates Jm was predicted by the equation *Jm = K*_*p*_ *× MW × S*, where K_p_ is the skin permeability obtained from QPlogK_p_, MW is molecular weight and S is the aqueous solubility obtained from QPlogS and Jm is the prediction of the maximum trans-dermal transport rate in μg cm^-2^hr^-1^. The computed values showed that the selected compounds possessed variation between 0 to1.5 μg cm^-2^hr^-1^ except 4 compounds with values >1.5 μg cm^-2^hr^-1^. However, none of these 4 compounds had a predicted value >100 μg cm^-2^hr^-1^.

### The prediction of plasma-protein binding

The distribution of drug through the blood stream and availability of drugs for its target depend on the ability of a compound/s to bind to the plasma protein such as lipoprotein, glycoprotein, human serum albumin, a, b, and c globulins which directly influence the drug efficacy. The binding of the drug to the plasma-proteins can greatly reduce the quantity of the drug, thereby reducing the rate of distribution of drug through general blood circulation. Therefore the less degree of plasma-protein binding is desirable for designing drug with more cell availability and cell membrane traverse/diffusion. The logKhsa has been computed for the estimation of plasma-protein binding to predict the tendency of the selected compounds to bind to the human serum albumin. The computed values show that all 13 compounds are in the recommended range (-1.5 to 1.5).

### The prediction of metabolism

The number of likely metabolic reactions is necessary for determining the level of accessibility of compounds to their target sites after entering into the blood stream. The average number of possible metabolic reactions of each compound has been predicted by using of the #meta parameter of QikProp. The results indicate that 84.61% of all 13 selected compounds, except cinnamic acid and dibenzoylmetane [31], possessed #meta values were within the recommended range of metabolic reaction 1−8. Cyclocurcumin [31] showed a tendency for 6 metabolic reactions due to the complexity of the molecule while cinnamic acid and dibenzoylmetane showed no reaction tendency.

### The prediction of blockage of human ether-a-go-go-related gene potassium (HERG K+) channel

Human ether-a-go-go related gene (HERG) is the target for testing the cardiac toxicity of drugable molecules [32] due to its role in the electrical activity of the heart during systolic and diastolic periods by encoding the potassium ion (K^+^) channel. This channel also has a modulating function in nervous system [33] and can be involved in disorders such as torsade de pointes (long QT syndrome) [34]. Thus, every molecule which blocks HERG K^+^ channel is potentially toxic for cardiac and nervous system and IC50 values is necessary to determine for prediction of toxicity of drugable molecules in drug designing [35]. By using Qikprop of Schrodinger 2011 the IC50 values of curcumin and analogs have been predicted and computed. The results indicate that 4 out of 13 selected compounds fall within the recommended range of log IC50 values for blockage of HERG K+ channels >-5. Among the selected compounds caffeic acid [30], isoeugenol, B-turmerone and ferulic acid showed logHERG values within the recommended range.

### Toxicity

Some important parameters for toxicity investigation have been predicted via online TOPKAT approaches of Accelrys Environmental Chemistry and Toxicology Workbench. The carcinogenicity of compounds have been predicted based on structural similarity between compounds and structures available in both the FDA (U.S. Food and Drug Administration) and NTP (National Toxicology Program) databases for male and female of rat and mouse (FR, FM, MR and MM). The predicted carcinogenicity for both rat and mouse in both sexes is based on the FDA database. B-turmerone [31] is severe carcinogen for both female and male mouse and rat with probability 0.246, 0.518, 0.347 and 0.393 for FM, MM, FR and MR respectively, but caffeic acid and ferulic acid are carcinogenic for MM with probability 0.290 and 0.236 and severe carcinogen for MR with probability 0.310 and 0.307 respectively but safe for FM and FR. Isoeugnol is non-carcinogenic for FR, FM and MR but carcinogenic for MM with probability 0.244. Based on NTP database isoeugenol, ferulic acid and B-turmerone are carcinogens for MM with probability 0.731, 0.792 and 0.604 and for FM with probability 0.592, 0.679 and 0.679 respectively. Caffeic acid is a non-carcinogen for FM but carcinogen for MM with probability 0.711. Caffeic acid, isoeugenol, B-turmerone and ferulic acid are safe for MR but isoeugenol and ferulic acid are carcinogenic for FR with probability 0.514 and 0.573 respectively. They are non-irritants for skin except B-turmerone which is a severe irritant for skin, but all are irritants for ocular and also showed skin sensitivity with probability range of 0.643 to 0.991. The Rat Oral LD50 values obtained were 2.026, 2.063, 1.681 and 1.842 gr/kg for isoeugenol, ferulic acid, caffeic acid and B-turmerone respectively, which were within the optimum prediction space (OPS) and indicated the high safety of these compounds.

According to WOE (weight of evidence for rodent carcinogenicity) except B-turmerone the other three compounds were devoid of carcinogenic potential in rodents with probability range of 0.478 to 0.490. Based on the DTP (developmental toxicity potential) model all the compounds were toxic and received a positive discriminant score and the probability range for the toxicity was 0.52 to 0.70. None of the compounds showed genotoxicity or mutagenicity potential. The EC50 values for Daphina Magna model are 0.466, 4.292, 6.315, and 4.036 mg/l for isoeugenol, ferulic acid, caffeic acid and B-turmerone respectively which are significant values. Except isoeugenol all other three compounds were degraded through an aerobic-biodegradablility pathway. Total quantitative prediction, including EC50, TD50, LD50, LC50, DTP, and other parameters which are widely used for prediction of toxicity of compounds are given in S1 Table.

Based on the results of ADME studies and toxicity profiles, three compounds, namely ferulic acid, caffeic acid and B-turmerone were shortlisted for further study of their therapeutic potential as cardiovascular safe anti-inflammatory agents.

### Docking calculations using Schrodinger 2011

**Fig 6:**
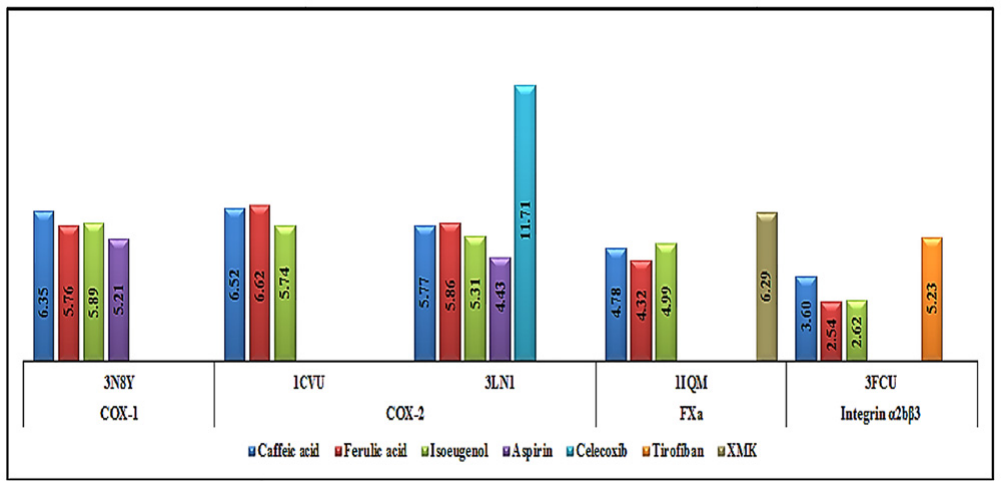
The affinity value comparison of caffeic acid, isoeugenol and ferulic acid with 4 common market drugs, aspirin, celecoxib, XMK and tirofiban in complex with 4 receptors, COX-1, COX-2, both PDB IDs, FXa, integrin α2bβ3.

The three compounds, caffeic acid, ferulic acid and isoeugenol, have been investigated for their binding efficacies to the receptors involved in inflammatory response and thrombosis using Glide. Fig 6 shows the affinity values of these three selected compounds along with four commonly available drugs, aspirin (non-selective NSAID for COX-1, COX-2) [36], celecoxib (selective NSAID for COX-2), XMK or 1-[[(1E)-2-(4-Chlorophenyl) Thenyl] Sulfonyl]-4-[[1-(4-Pyridinyl)-4-Piperidinyl] Methyl] Piperazine (specific drug for FXa) and Tirofiban (specific integrin α2bβ3) in complex with four receptors COX-1, COX-2 (both PDB IDs:1CUV and 3LN1), FXa and integrin α2bβ3 which were indicated as kcal/mol.

Caffeic acid and ferulic acid have similar values for COX-2 inhibition in both substrate and inhibitor binding sites and have higher affinity values as compared to isougenol and aspirin. However, their inhibitory potential is significantly lesser than celecoxib. But caffeic acid had higher docking scores than ferulic acid in complex with COX-1, FXa and integrin α2bβ3. caffeic acid can be a good competitor for aspirin in inhibition of COX-1 and COX-2 and inhibit prostaglandins and TXA2 synthase. It also has potential to moderately inhibit FXa and integrin α2bβ3 enzymes which prevent platelet aggregation and thrombosis. Table 3 contains the affinity values and MMGBSA of the all the analogs of curcumin that were studied with each of the four receptors namely COX-1, COX-2 (both PDB IDs), FXa and integrin α2bβ3.

### Interaction between curcumin and its analogs and residues in receptors

**Fig 7:**
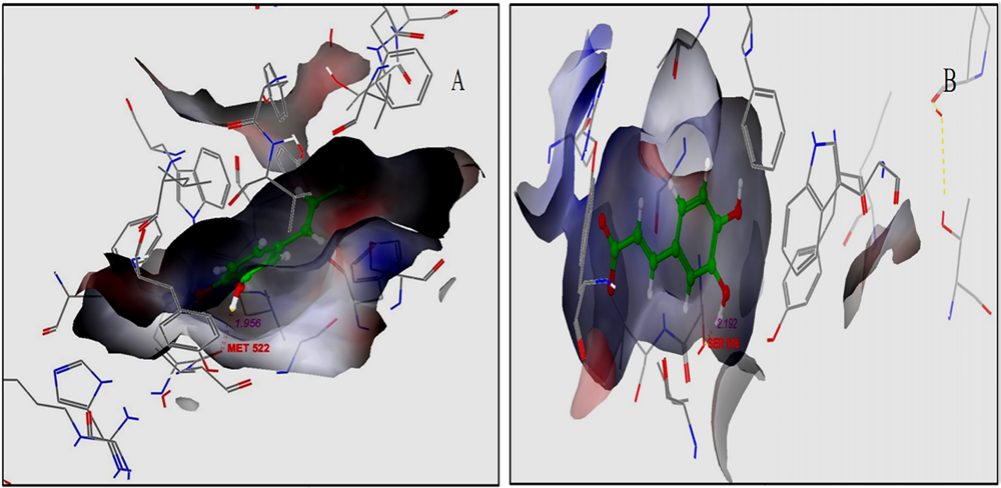
Interaction caffeic acid with active sites of COX-2. (A) Caffeic acid in the binding pocket of COX-2 with PDB ID: 1CVU. (B) Caffeic acid in the binding pocket of COX-2 with PDB ID: 3LN1.

The interaction of caffeic acid, a phytochemical of biological origin, with COX-2 with PDB IDs, 1CVU and 3LN1 is shown in Fig 7A and 7B respectively. Caffeic acid interacts through a hydrogen bond with MET522 having bond distance of 1.984 angstrom in the binding pocket of 1CVU. The other amino acid residues present in the binding pocket of 1CVU are LEU534, SER530, MET522, LEU384, ALA527, TYR385, GLY526, PHE381, LEU352, VAL349, PHE518, PHE209, PHE205, TYR384, VAL344, VAL523, TRP387 and TRY385 along with residue 3046, 3084, 3137, 3173, 3519, 3648, 3649, and 3755 which interact with water molecules. In Fig 7B, caffeic acid forms 1 hydrogen bond with SER516 having a bond distance of 2.192 angstrom in the 3LN1 binding pocket. The other amino acid residues present in the binding pocket of 3LN1 are Gly512, Ala513, Met508, Val509, Tyr341, Phe504, Leu338, Val335, Trp373, ser516, arg106, try334, leu517 and Try371 and the two water molecules interact with amino acid residues 15 and 613.

**Fig 8:**
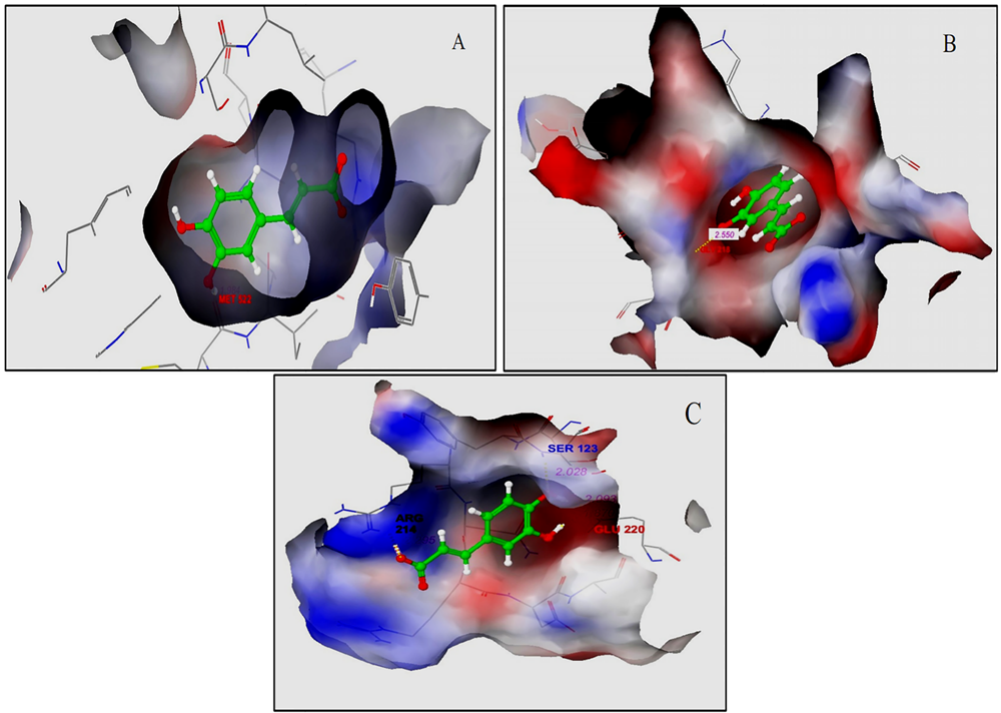
3D structures of caffeic acid in the binding pocket of(A) COX-1with PDB ID: 1DIY, (B) FXa with PDB ID: 1QIM, and (C) integrin α2bβ3 with PDB ID: 3FCU.

The interactions of caffeic acid with 3 receptors, COX-1, integrin α2bβ3 and FXa, are shown in Fig 8A, 8B and 8C respectively. The interaction of caffeic acid with COX-1 was through one hydrogen bond with MET522 having bond distance of 1.984 angstrom. The other amino acid residues present in the binding pocket of COX-1 were VAL349, LEU352, SER353, TYR355, TYR385, TRP387, MET522, ILE523, GLY526, ALA527, SER530 and LEU531 and water molecules attached to residue 27, 609 and 617. Caffeic acid binds to the active site of FXa through one hydrogen bond with GLY218 having bond distance of 2.550 angstrom. The other amino acid residues present in the binding pocket of FXa are ASP198, GLY216, Ala190, Val213, Try228, Gly226, Ile227, Trp215, Thr98, Glu97, Phe174, Cys220, Cys191 and Glu 192 and water molecules attached to residue 532 on the protein surface through hydrogen bond. The complex of caffeic acid-integrin α2bβ3 formed 4 hydrogen bonds with Arg214 and Ser123 with bond distances 2.295 and 2.028 angstrom respectively, and two hydrogen bonds with bond distances 2.093 and 1.870 angstrom with GLU220. The other amino acid residues present in the binding pocket of COX-1 were ser123, Asn215, Arg214, Ser121, Glu220, Asp217, Arg216, Tyr166, TRY122 and ALA218 without any water molecule attached to residues in the binding site.

## Conclusion

Drug target identification is becoming an overly time consuming process and in many cases produces inefficient results due to failure of conventional approaches like *in vivo* and *in vitro* to investigate large scale data. Sophisticated *in silico* approaches has given a tremendous opportunity to pharmaceutical companies to identify new potential drug targets which in turn affect the success and time of performing clinical trials for discovering new drug targets. The main goal of this work is *in silico* study for drug discovery process with emphasis on identifying drug targets for cardiovascular-safe anti-inflammatory. Various analogs that were phytochemical in origin were studied to assess their ADME and toxicity properties. Out of the 39 analogs studied caffeic acid was found to have remarkable interactions with the proteins involved in inflammatory response as compared to commonly available drugs such as asprin and celecoxib. Therefore we have been able to identify caffeic acid as a potential therapeutic agent having anti-inflammatory potential due to its interacting with COX-1 and COX-2. Thrombosis have been reported following the use of the drug celecoxib, but caffeic acid was seen to inhibit COX- 1, FXa and integrin α2bβ3, thereby being safe for the cardiovascular system.

## Supporting Information

S1 File Minimal Dataset. (DOC)

S1 Table Quantitative toxicity profiling of all Curcumin and its analogs. (XLS)

S2 Table ADME profiling of all compounds. (DOC)
