# Supplementary material for: Drug Targets for Cardiovascular-Safe Anti-Inflammatory: In Silico Rational Drug Studies
Source: PLoS One. 2016 Jun 3;11(6):e0156156. doi: 10.1371/journal.pone.0156156 (PMC4892653; doi:10.1371/journal.pone.0156156)
Supplement: S1 File — (DOC) [file pone.0156156.s001.doc]

**Minimal Dataset**

In the present study, we performed *in silico* studies to quantitatively scrutinize the molecular interaction of curcumin and its structural analogs with COX-2, COX-1, FXa and Integrin αIIbβIII to investigate their therapeutic potential as a cardiovascular-safe anti-inflammatory medicine (CVSAIM). The results of both ADMET and docking study indicated that out of all the 39 compounds studied, Caffeic acid had remarkable interaction with proteins involved in inflammatory response. It was also found to inhibit the proteins that are involved in thrombosis, thereby, having the potential to be developed as therapeutic agent.
