# Supplementary material for: Drug Targets for Cardiovascular-Safe Anti-Inflammatory: In Silico Rational Drug Studies
Source: PLoS One. 2016 Jun 3;11(6):e0156156. doi: 10.1371/journal.pone.0156156 (PMC4892653; doi:10.1371/journal.pone.0156156)
Supplement: S2 Table — (DOC) [file pone.0156156.s003.doc]

**S2.1 Table. ADME profiling**

| **Title** | **CNS** | **SASA** | **FOSA** | **FISA** | **PISA** | **volume** | **IP(eV)** | **EA(eV)** | **#metab** | **glob** | **Jm** |
| --- | --- | --- | --- | --- | --- | --- | --- | --- | --- | --- | --- |
| **Enol** |  |  |  |  |  |  |  |  |  |  |  |
| Bisdemethoxycurcumin | -2 | 627.61 | 84.93 | 188.92 | 353.76 | 1054.68 | 8.90 | 0.73 | 3 | 0.80 | 0.05 |
| BJC003 | -1 | 669.26 | 260.59 | 87.41 | 321.27 | 1135.02 | 9.28 | 0.67 | 3 | 0.79 | 0.06 |
| BJC004 | -2 | 732.59 | 234.80 | 255.66 | 242.13 | 1263.84 | 10.15 | 1.51 | 5 | 0.77 | 0.00 |
| BJC005 | -2 | 762.09 | 251.22 | 325.13 | 185.75 | 1339.17 | 0.00 | 0.00 | 7 | 0.77 | 0.00 |
| Compound I | -2 | 725.21 | 238.47 | 218.71 | 268.03 | 1250.12 | 8.02 | 0.71 | 4 | 0.77 | 0.01 |
| Compound II | -2 | 826.06 | 221.27 | 117.29 | 487.50 | 1465.99 | 7.71 | 1.09 | 7 | 0.76 | 0.16 |
| Compound III | -2 | 883.17 | 259.78 | 282.65 | 340.75 | 1623.88 | 8.86 | 1.52 | 8 | 0.76 | 0.00 |
| Curcumino-2-phenylenediimine | -2 | 1277.77 | 551.11 | 260.99 | 465.67 | 2500.49 | 8.73 | 0.13 | 18 | 0.70 | 0.00 |
| Curcumino-iminobenzene | -2 | 809.89 | 238.50 | 124.78 | 446.61 | 1442.05 | 8.67 | 1.02 | 4 | 0.76 | 0.02 |
| Curcomin | -2 | 693.80 | 239.36 | 197.00 | 257.43 | 1200.80 | 8.81 | 1.01 | 5 | 0.79 | 0.01 |
| Demethoxycurcumin | -2 | 646.63 | 144.40 | 192.88 | 309.35 | 1117.50 | 9.11 | 1.02 | 4 | 0.81 | 0.04 |
| **Keto** |  |  |  |  |  |  |  |  |  |  |  |
| Ar-Curcumene | 2 | 521.89 | 395.62 | 0.00 | 126.27 | 879.54 | 9.17 | -0.34 | 5 | 0.85 | 0.00 |
| Ar-Turmerone | 0 | 501.54 | 337.86 | 37.27 | 126.42 | 855.09 | 9.22 | 0.01 | 5 | 0.87 | 0.86 |
| B-Turmerone | 0 | 508.59 | 360.38 | 40.64 | 107.57 | 875.99 | 9.08 | 0.19 | 5 | 0.87 | 0.86 |
| Bisdemethoxycurcumin | -2 | 619.25 | 67.79 | 193.73 | 357.73 | 1045.23 | 8.84 | 1.09 | 3 | 0.80 | 0.05 |
| Caffeic acid | -2 | 392.03 | 29.49 | 216.65 | 145.89 | 612.16 | 0.00 | 0.00 | 2 | 0.89 | 0.25 |
| Capsaicin | 0 | 620.54 | 465.75 | 18.70 | 136.09 | 1065.64 | 8.96 | -0.24 | 5 | 0.81 | 2.10 |
| Cassumuin A | -2 | 916.35 | 374.38 | 168.33 | 373.63 | 1644.58 | 8.63 | 0.81 | 7 | 0.74 | 0.00 |
| Cassumuin B | -2 | 969.13 | 475.56 | 180.43 | 313.15 | 1740.92 | 8.49 | 0.88 | 8 | 0.72 | 0.00 |
| Cinnamic acid | -1 | 368.62 | 29.51 | 114.99 | 224.13 | 568.11 | 0.00 | 0.00 | 0 | 0.90 | 6.34 |
| Curcumin | -2 | 685.98 | 259.76 | 160.53 | 265.69 | 1193.77 | 9.07 | 0.89 | 5 | 0.79 | 0.09 |
| Curcumin sulphate | -2 | 777.04 | 257.27 | 278.34 | 238.85 | 1349.71 | 0.00 | 0.00 | 4 | 0.76 | 0.00 |
| Cyclocurcumin | -2 | 669.69 | 243.49 | 155.67 | 270.54 | 1174.12 | 8.82 | 1.11 | 6 | 0.80 | 0.00 |
| Demethoxycurcumin | -2 | 648.19 | 160.59 | 181.23 | 306.37 | 1116.96 | 8.96 | 0.90 | 4 | 0.80 | 0.06 |
| Dibenzoylmethane | 0 | 479.16 | 13.65 | 73.86 | 391.65 | 790.54 | 9.23 | 0.65 | 0 | 0.86 | 5.79 |
| Dicaffeoylmethane | -2 | 650.21 | 75.65 | 285.00 | 289.56 | 1101.09 | 0.00 | 0.00 | 5 | 0.79 | 0.00 |
| Dihydrocurcumin | -2 | 693.70 | 309.85 | 149.40 | 234.46 | 1216.13 | 8.85 | 0.84 | 7 | 0.79 | 0.08 |
| Ferulic acid | -2 | 420.21 | 122.13 | 169.02 | 129.07 | 669.34 | 0.00 | 0.00 | 2 | 0.88 | 0.48 |
| Gingerol | -1 | 618.59 | 463.88 | 59.84 | 94.87 | 1064.23 | 8.75 | -0.31 | 5 | 0.81 | 0.54 |
| Hexa hydrocurcumin | -2 | 716.76 | 349.34 | 161.39 | 206.03 | 1253.75 | 8.82 | 0.01 | 9 | 0.78 | 0.01 |
| Hexa hydrocurcuminol | -2 | 691.49 | 357.30 | 147.74 | 186.45 | 1236.12 | 8.81 | -0.20 | 8 | 0.81 | 0.25 |
| Hydrazinocurcumins | -2 | 675.20 | 206.68 | 145.64 | 322.88 | 1149.17 | 8.36 | 0.68 | 4 | 0.79 | 0.00 |
| Isoeugenol | 0 | 362.72 | 153.91 | 49.38 | 159.44 | 573.66 | 8.73 | 0.20 | 2 | 0.92 | 58.62 |
| Methylcurcumin | -2 | 746.03 | 410.58 | 88.60 | 246.85 | 1309.59 | 8.95 | 1.05 | 5 | 0.78 | 0.54 |
| Methylthiomethylcurcumin | -2 | 792.08 | 469.17 | 168.52 | 114.31 | 1484.75 | 8.66 | 0.84 | 7 | 0.79 | 0.01 |
| Rosmarinic acid | -2 | 647.78 | 70.67 | 333.47 | 243.64 | 1139.07 | 0.00 | 0.00 | 6 | 0.81 | 0.00 |
| Sodiumcurcuminate | -2 | 687.75 | 247.55 | 178.30 | 261.90 | 1195.47 | 8.88 | 0.90 | 5 | 0.79 | 0.04 |
| Tetra hydrocurcumin | -2 | 695.28 | 331.38 | 127.39 | 236.51 | 1221.96 | 8.71 | 0.07 | 8 | 0.80 | 0.04 |
| Trimethylcurcumin | -1 | 745.09 | 431.92 | 67.13 | 246.04 | 1329.88 | 8.77 | 1.03 | 5 | 0.78 | 1.44 |

**CNS**: central nervous system activity-2, ,-1, 0, 1, 2: -2=completely inactive, -1=very low activity, 0=low activity, 1=medium activity, 2= completely active, 3=high, **Jm**: maximum transdermal transport rate, **SASA**: Total solvent accessible surface area;300-1000, **FOSA**: Hydrophobic component;0-750, **FISA**: Hydrophilic component;7-330, **Volume**: Total solvent-accessible volume;500-2000, **PISA**: π (carbon and attached hydrogen) component of the SASA;7-200, **Glob**: Globularity descriptor;0.75-0.95, **IP(eV):** PM3 calculated ionization potential; 7.9 – 10.5, **EA(eV):** PM3 calculated electron afﬁnity; –0.9 – 1.7, **Metab**: Number of likely metabolic reactions; 1 – 8.

**S2.2 Table. ADME profiling**

| **Title** | **QPlog**  **S** | **CIQPlog**  **S** | **QPlog**  **HERG** | **QPP**  **Caco** | **QPlog**  **BB** | **QPP**  **MDCK** | **QPlog**  **Kp** | **QPlog**  **Khsa** | **HOA** | **RF** | **RT** |
| --- | --- | --- | --- | --- | --- | --- | --- | --- | --- | --- | --- |
| **Enol** |  |  |  |  |  |  |  |  |  |  |  |
| Bisdemethoxycurcumin | -4.04 | -4.00 | -6.48 | 160.10 | -2.02 | 68.29 | -2.80 | -0.05 | 3 | 0 | 0 |
| BJC003 | -5.50 | -4.40 | -6.52 | 1468.98 | -0.86 | 749.68 | -1.23 | 0.50 | 3 | 0 | 0 |
| BJC004 | -5.33 | -5.39 | -6.38 | 37.28 | -2.90 | 14.14 | -4.42 | 0.26 | 3 | 0 | 0 |
| BJC005 | -4.53 | -5.62 | -6.12 | 8.18 | -3.93 | 2.74 | -5.51 | -0.14 | 2 | 1 | 2 |
| Compound I | -4.38 | -5.20 | -6.47 | 83.53 | -2.78 | 33.81 | -3.17 | -0.02 | 2 | 0 | 0 |
| Compound II | -6.04 | -7.01 | -7.81 | 764.92 | -1.77 | 370.30 | -0.43 | 0.52 | 1 | 1 | 2 |
| Compound III | -6.69 | -8.44 | -7.10 | 20.68 | -3.75 | 7.48 | -3.90 | 0.78 | 1 | 2 | 3 |
| Curcumino-2-phenylenediimine | -9.35 | -12.62 | -8.65 | 33.19 | -4.82 | 12.46 | -1.81 | 1.39 | 1 | 4 | 2 |
| Curcumino-iminobenzene | -6.45 | -7.20 | -7.51 | 649.50 | -1.77 | 310.29 | -0.81 | 0.78 | 1 | 1 | 1 |
| Curcomin | -4.37 | -4.62 | -6.20 | 134.19 | -2.28 | 56.43 | -3.09 | -0.03 | 2 | 0 | 0 |
| Demethoxycurcumin | -3.97 | -4.31 | -6.16 | 146.83 | -2.08 | 62.20 | -2.93 | -0.05 | 3 | 0 | 0 |
| **Keto** |  |  |  |  |  |  |  |  |  |  |  |
| Ar-Curcumene | -6.97 | -6.97 | -4.55 | 9906.04 | 0.98 | 5899.29 | -0.69 | 1.00 | 1 | 1 | 1 |
| Ar-Turmerone | -4.02 | -3.10 | -4.26 | 4390.44 | -0.01 | 2448.08 | -1.38 | 0.40 | 3 | 0 | 0 |
| B-Turmerone | -3.99 | -2.95 | -4.14 | 4078.42 | -0.10 | 2260.58 | -1.41 | 0.41 | 3 | 0 | 0 |
| Bisdemethoxycurcumin | -3.90 | -4.00 | -6.40 | 144.14 | -2.04 | 60.97 | -2.87 | -0.07 | 3 | 0 | 0 |
| Caffeic acid | -1.34 | -1.84 | -2.21 | 22.13 | -1.56 | 10.23 | -4.52 | -0.80 | 2 | 0 | 1 |
| Capsaicin | -4.41 | -2.90 | -3.83 | 5079.75 | -0.05 | 3794.09 | -0.71 | 0.09 | 3 | 0 | 0 |
| Cassumuin A | -7.31 | -7.60 | -7.60 | 250.95 | -2.48 | 111.01 | -1.77 | 0.79 | 1 | 2 | 2 |
| Cassumuin B | -7.80 | -7.90 | -7.52 | 192.72 | -2.77 | 83.45 | -2.11 | 0.85 | 1 | 2 | 2 |
| Cinnamic acid | -1.66 | -1.78 | -2.41 | 203.75 | -0.56 | 112.72 | -2.56 | -0.51 | 3 | 0 | 0 |
| curcumin | -4.24 | -4.62 | -6.14 | 297.56 | -1.86 | 133.46 | -2.39 | -0.04 | 3 | 0 | 0 |
| Curcumin sulphate | -4.20 | -4.75 | -4.75 | 5.75 | -3.49 | 2.47 | -4.46 | -0.62 | 2 | 0 | 1 |
| Cyclocurcumin | -5.17 | -5.31 | -6.00 | 330.89 | -1.47 | 149.69 | -2.76 | 0.35 | 3 | 0 | 0 |
| demethoxycurcumin | -4.00 | -4.31 | -6.18 | 189.36 | -1.97 | 81.88 | -2.72 | -0.06 | 3 | 0 | 0 |
| Dibenzoylmethane | -3.57 | -3.74 | -5.62 | 1974.58 | -0.40 | 1032.11 | -1.02 | 0.23 | 3 | 0 | 0 |
| Dicaffeoylmethane | -3.47 | -3.96 | -6.26 | 19.65 | -3.18 | 7.07 | -4.60 | -0.37 | 2 | 0 | 1 |
| Dihydrocurcumin | -4.37 | -4.66 | -5.98 | 379.46 | -1.74 | 173.57 | -2.30 | 0.02 | 3 | 0 | 1 |
| Ferulic acid | -1.91 | -2.15 | -2.27 | 62.62 | -1.19 | 31.49 | -3.70 | -0.61 | 3 | 0 | 0 |
| Gingerol | -4.48 | -3.82 | -4.98 | 2681.62 | -0.70 | 1436.80 | -1.23 | 0.37 | 3 | 0 | 0 |
| Hexa hydrocurcumin | -5.02 | -5.22 | -6.00 | 292.06 | -1.97 | 130.80 | -2.52 | 0.30 | 3 | 0 | 1 |
| Hexa hydrocurcuminol | -3.94 | -4.83 | -5.57 | 393.42 | -1.80 | 180.48 | -2.24 | -0.04 | 3 | 0 | 1 |
| Hydrazinocurcumins | -5.84 | -5.69 | -6.54 | 411.89 | -1.49 | 189.66 | -2.30 | 0.67 | 3 | 0 | 1 |
| Isoeugenol | -1.57 | -1.97 | -3.58 | 3370.25 | -0.01 | 1839.46 | -1.58 | -0.25 | 3 | 0 | 0 |
| Methylcurcumin | -4.74 | -4.96 | -6.34 | 1431.21 | -1.14 | 728.86 | -1.13 | 0.04 | 3 | 0 | 0 |
| Methylthiomethylcurcumin | -5.23 | -6.61 | -5.40 | 249.94 | -2.08 | 183.25 | -2.69 | 0.36 | 1 | 0 | 1 |
| Rosmarinic acid | -3.28 | -4.49 | -3.89 | 1.73 | -3.51 | 0.65 | -5.66 | -0.49 | 1 | 0 | 1 |
| Sodiumcurcuminate | -4.27 | -4.62 | -6.15 | 201.86 | -2.06 | 87.74 | -2.73 | -0.04 | 2 | 0 | 0 |
| Tetra hydrocurcumin | -5.12 | -5.53 | -5.98 | 613.54 | -1.50 | 291.76 | -1.88 | 0.44 | 3 | 0 | 1 |
| Trimethylcurcumin | -4.72 | -5.26 | -6.19 | 2287.01 | -0.89 | 1209.70 | -0.74 | 0.10 | 3 | 0 | 0 |

**QPlogS**: prediction aqueous solubility level; recommended range -6.5<x<0.5,**CIQPlogS**:Conformation-independent predicted aqueous solubility; -6.5<x<0.5,**QPlogHERG**: Predicted IC50 value for blockage of HERG K+ channels; <-5= concern, **QPPCaco**: Predicted apparent gut-blood barrier permeability; <25=poor, >500=great, **QPlogBB**: Predicted brain/blood partition coefﬁcient; –3.0 – 1.2, **QPPMDCK**: Predicted apparent MDCK cell permeability; <25=poor,>500= great, **QPlogKp**: Predicted skin permeability; range= -8<x<-1**, QPlogKhsa**: Prediction of binding to human serum albumin; –1.5 – 1.5,**HOA**: human oral absorption level;1, 2, 3; 1=low, 2=medium,**RF**: the number of violations of Lipinski’s rule of five, **RT:** the number of violations of Jorgensen’s rule of three.
